# Nomogram for prediction of level 2 axillary lymph node metastasis in proven level 1 node-positive breast cancer patients

**DOI:** 10.18632/oncotarget.20395

**Published:** 2017-08-22

**Authors:** Yanlin Jiang, Hong Xu, Hao Zhang, Xunyan Ou, Zhen Xu, Liping Ai, Lisha Sun, Caigang Liu

**Affiliations:** ^1^ Department of Breast Surgery, Shengjing Hospital of China Medical University, Shenyang, Liaoning Province, 110004, China; ^2^ Department of Breast Surgery, Cancer Hospital of China Medical University (Liaoning Cancer Hospital & Institute), Shenyang, Liaoning Province, 110042, China; ^3^ Department of Breast Disease and Reconstruction Center, Breast Cancer Key Lab of Dalian, the Second Hospital of Dalian Medical University, Dalian, 116027, China; ^4^ Department of Surgical Oncology, The First Hospital of China Medical University, Shenyang, 110013, China

**Keywords:** breast cancer, level 2 axillary lymph node metastasis, level 1 axillary lymph node metastasis, nomogram

## Abstract

**Background:**

The current management of the axilla in level 1 node-positive breast cancer patients is axillary lymph node dissection regardless of the status of the level 2 axillary lymph nodes. The goal of this study was to develop a nomogram predicting the probability of level 2 axillary lymph node metastasis (L-2-ALNM) in patients with level 1 axillary node-positive breast cancer.

**Materials and Methods:**

We reviewed the records of 974 patients with pathology-confirmed level 1 node-positive breast cancer between 2010 and 2014 at the Liaoning Cancer Hospital and Institute. The patients were randomized 1:1 and divided into a modeling group and a validation group. Clinical and pathological features of the patients were assessed with uni- and multivariate logistic regression. A nomogram based on independent predictors for the L-2-ALNM identified by multivariate logistic regression was constructed.

**Results:**

Independent predictors of L-2-ALNM by the multivariate logistic regression analysis included tumor size, Ki-67 status, histological grade, and number of positive level 1 axillary lymph nodes. The areas under the receiver operating characteristic curve of the modeling set and the validation set were 0.828 and 0.816, respectively. The false-negative rates of the L-2-ALNM nomogram were 1.82% and 7.41% for the predicted probability cut-off points of < 6% and < 10%, respectively, when applied to the validation group.

**Conclusions:**

Our nomogram could help predict L-2-ALNM in patients with level 1 axillary lymph node metastasis. Patients with a low probability of L-2-ALNM could be spared level 2 axillary lymph node dissection, thereby reducing postoperative morbidity.

## INTRODUCTION

Owing to its increasing incidence, breast cancer has become one of the most common malignancies, accounting for 15% of all cancer-related deaths [1–3]. Clear axillary lymph node metastasis greatly influences individualized treatment decision making and has become one of the most important prognostic factors in patients with breast cancer [4–6].

For patients clinically diagnosed as having a level 1 axillary lymph node metastasis (L-1-ALNM), the standard surgical procedure includes primary tumor resection and axillary lymph node dissection (ALND; including level 1 and 2 axillary lymph nodes), regardless of whether the level 2 axillary lymph node is positive or negative for malignancy. The ACOSOG Z0011 trial [7] demonstrated axillary lymph node dissection could not benefit them compared with sentinel lymph node biopsy for patients with 12 positive sentinel lymph nodes. In other words, extensive negtive axillary lymph node dissection surgery could not decrease local recurrence rate and improve patient survival in breast cancer patients. However, sentinel lymph node biopsy (SLNB) is not a reasonable and reliable treatment, for the patients with preoperatively confirmed lymph node metastasis. Therefore, level 1 axillary lymph node dissection could be a better option for patients with positive level 1 axillary lymph nodes but without L-2-ALNM. Several retrospective studies showed that less negtive axillary lymph node dissection decreased the incidence of short- and long-term postoperative complications such as limited range of shoulder mobility, lymphedema, and upper arm numbness. Thus, omission of L-2-ALND provides patients with better quality of life than performing ALND [8–10]. These findings suggest that a nomogram should be established urgently to evaluate the level 2 axillary lymph node status in patients with level 1 node-positive breast cancer and to identify those in whom L-2-ALND can be omitted.

In some studies, a perfect nomogram has been shown to accurately assess the risk of lymph node metastasis and provide a reliable basis for clinicians to make decisions about breast cancer treatment [11, 12]. Currently, no well-designed nomogram is available to evaluate the probability of L-2-ALNM in patients with level 1 axillary lymph node metastasis. Therefore, our present research was intended to identify possible predictors of L-2-ALNM and to construct a model to calculate the risk of L-2-ALNM in patients with level 1 axillary node-positive breast cancer, which would increase the accuracy of surgical decision making. With accurate nomogram predictions, patients with a low risk of L-2-ALNM can avoid undergoing L-2-ALND.

## RESULTS

### Patient characteristics

Our study included 974 patients randomized 1:1 into a modeling set (*n* = 487) and a validation set (*n* = 487). Table 1 depicted the clinical and pathological characteristics of the modeling and validation groups. The L-2-ALNM rates of both groups were the same (27.9%, 136/487). The clinicopathological characteristics of the patients did not differ significantly between the two groups (*P* > 0.05) in our study population. The univariate analysis of the modeling was shown in Table 2.

**Table 1:** Comparison between modeling group and validation group by clinicopathological characteristics

Variables	Modeling No. (%)	Validation No. (%)	*P*-value
No. of Patients	487 (100%)	487 (100%)	
L-2-ALNM			1.000
Yes	136 (27.9)	136 (27.9)	
No	351 (72.1)	351 (72.1)	
Age (year)			0.605
≤ 45	119 (24.4)	126 (25.9)	
> 45	368 (75.6)	361 (74.9)	
Menopausal status			0.305
Premenopausal	237 (48.7)	253(52.0)	
Postmenopausal	250 (51.3)	234 (48.0)	
Tumor size			0.991
T1 ≤ 2 cm	151 (31.0)	150 (30.8)	
2 cm < T2 ≤ 3 cm	266 (54.6)	265 (54.4)	
3 cm < T3 ≤ 5 cm	65 (13.3)	66 (13.6)	
T4 > 5 cm	5 (1.0)	6 (1.2)	
ER status			0.937
Negative	100 (20.5)	99 (20.3)	
Positive	387(79.5)	388 (79.7)	
PR status			0.777
Negative	138 (28.3)	142 (29.2)	
Positive	349 (71.7)	345 (70.8)	
Her-2 status			0.840
Negative	433(88.9)	431(88.5)	
Positive	54 (11.1)	56 (11.5)	
Ki-67 status			0.797
≤ 20	(47.2)	234 (48.0)	
> 20	257 (52.8)	253 (52.0)	
No. of PL-1-ALN			1.000
1	187 (38.4)	187 (38.4)	
2	103 (21.1)	102 (20.9)	
3	54 (11.1)	55 (11.3)	
4	43 (8.8)	43 (8.8)	
≥ 5	100 (20.5)	100 (20.5)	
Tumor location			0.089
UOQ	251 (51.5)	284 (58.3)	
LOQ	48 (9.9)	47 (9.7)	
LIQ	36 (7.4)	35 (7.2)	
UIQ	72 (14.8)	69 (14.2)	
Central	80 (16.4)	52 (10.7)	

Abbreviations: UOQ, upper outer quadrant; UIQ,upper inner quadrant; LOQ, lower outer quadrant; LIQ, lower inner quadrant.

**Table 2:** Univariate analysis analysis of different variables predicting L-2-ALNM of the modeling set

Variables	No L-2-ALNM No. (%)	L-2-ALNM No. (%)	*P*-value
No. of Patients	351 (100%)	136 (100%)	
Age (year)			0.957
≤ 45	86 (24.5)	33 (24.3)	
> 45	265 (75.5)	103 (75.7)	
Menopausal status			0.811
Premenopausal	172 (49.0)	65 (47.8)	
Postmenopausal	179 (51.0)	71 (52.2)	
Tumor size			< 0.001
T1 ≤ 2 cm	124 (35.3)	27 (19.9)	
2 cm < T2 ≤ 3 cm	193 (55.0)	73 (53.7)	
3 cm < T3 ≤ 5 cm	34 (9.7)	31 (22.8)	
T4 > 5 cm	0 (0.0)	5 (3.7)	
Histological grade			< 0.001
1	37 (10.5)	4 (2.9)	
2	287 (81.8)	102 (75.0)	
3	27 (7.7)	30 (22.1)	
ER status			0.077
Negative	65(18.5)	35 (25.7)	
Positive	286 (81.5)	101 (74.3)	
PR status			0.221
Negative	94 (26.8)	44 (32.4)	
Positive	257 (73.2)	92 (67.6)	
Her-2 status			0.011
Negative	320 (91.2)	113 (98.1)	
Positive	31 (8.8)	23 (16.9)	
Ki-67 status			< 0.001
≤ 20	172(49.0)	65 (47.8)	
> 20	179 (51.0)	71 (52.2)	
No. of PL-1-ALN			< 0.001
1	171 (48.7)	16 (11.8)	
2	87 (23.9)	19(14.0)	
3	35 (10.0)	19 (14.0)	
4	29 (8.3)	14 (10.3)	
≥ 5	32 (9.1)	68 (50.0)	
Tumor location			0.978
UOQ	180 (51.3)	71 (52.2)	
LOQ	36 (10.3)	12 (8.8)	
LIQ	25 (7.1)	11 (8.1)	
UIQ	53 (15.1)	19 (14.0)	
Central	57 (16.2)	23 (16.9)	

### Predictors of L-2-ALNM

After the univariate analysis, the following statistically significant (*P* < 0.05) variables were entered in the multivariate model: tumor size, Ki-67 status, HER-2 status, histological grade, and number of PL-1-ALN. The multivariate logistic regression analysis revealed that larger tumor size, positive Ki-67 status, higher histological grade, and greater number of PL-1-ALN remarkably correlated with greater risk of L-2-ALNM (Table 3).

**Table 3:** Results of multivariate logistic regression testing the association of each variable with L-2-ALNM

Variables	Coefficient	SE.	Wald value	*P* value	OR	95% CI	
						Lower	Upper
Histological grade							
1				0.055			
2	0.922	0.590	2.444	0.069	2.514	0.791	7.984
3	1.506	0.659	5.224	0.118	4.508	1.239	16.399
Tumor size	0.552	0.182	9.197	0.002	1.736	1.215	2.479
Ki-67 status	0.728	0.252	8.357	0.004	2.072	1.264	3.395
No. of PL-1-ALN	0.683	0.079	74.779	< 0.001	1.979	1.696	2.311
Her-2 status	0.017	0.361	0.002	0.961	1.018	0.502	2.063
Constant	−5.314	0.723	54.039	< 0.001	0.005		

### Construction of the nomogram

To evaluate the preoperative probability of L-2-ALNM based on the results of the multivariate logistic regression analyses, the following equation was developed: ln (*p*/*1* − *p*) = 0.683 × *a* + 0.552 × *b* + 0.728 × *c* + 0.922 × d1 + 1.506 × d2 − 5.314, where *p* represents the probability of L-2-ALNM, *a* represents the number of PL-1-ALN (1 if 1 positive level 1 axillary lymph node, 2 if 2 positive level 1 axillary lymph nodes, 3 if 3 positive level 1 axillary lymph nodes, 4 if 4 positive level 1 axillary lymph nodes, and 5 if > 5 positive level 1 axillary lymph nodes), *b* represents the tumor size (1 if T1, 2 if T2, 3 if T3, and 4 if T4), *c* represents Ki-67 status (0 if negative and 1 if positive), *d*1 represents histological grade 2 (0 if grade 2 [G1] or grade 3 [G3], and 1 if grade [G2]), *d*2 represents histological grade 3 (0 if G1 or G2, and 1 if G3).

**Figure 1:**
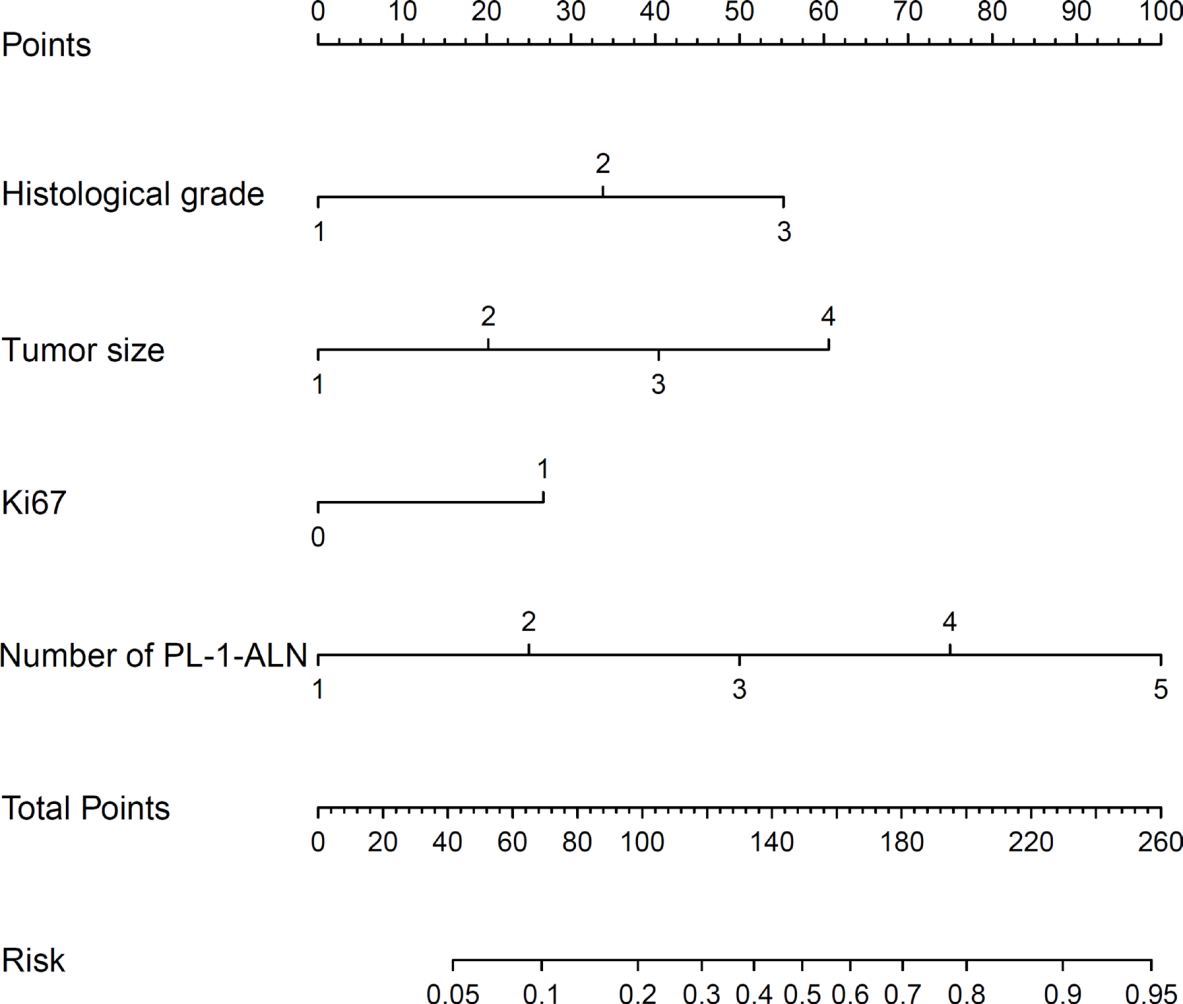
Nomogram for predicting the probability of L-2-ALNM The nomogram has seven rows. The first row is the point assignment for each variable. For each individual patient, each variable is assigned a point value in accordance with the clinicopathological characteristics by delineating a vertical line between the exact variable value and the points line. Thereafter, the Total Points (row 6) can be obtained by summing all of the assigned points for the four variables. Finally, the predictive probability of axillary metastasis can be acquired by drawing a vertical line between Total Points and Risk (the final row).

A predictive model presented in the form of a nomogram based on the results of the multivariate logistic regression analysis was established (Figure 1). The nomogram consisted of 7 rows. The first row was the point assignment for each variable, and the second to fifth rows were the four variables of the L-2-ALNM nomogram. The scores of the four variables were added to the total score presented on the scale in row 6, which corresponds to the prediction of the risk of L-2-ALNM in row 7.

### Validation of the L-2-ALNM nomogram

**Figure 2:**
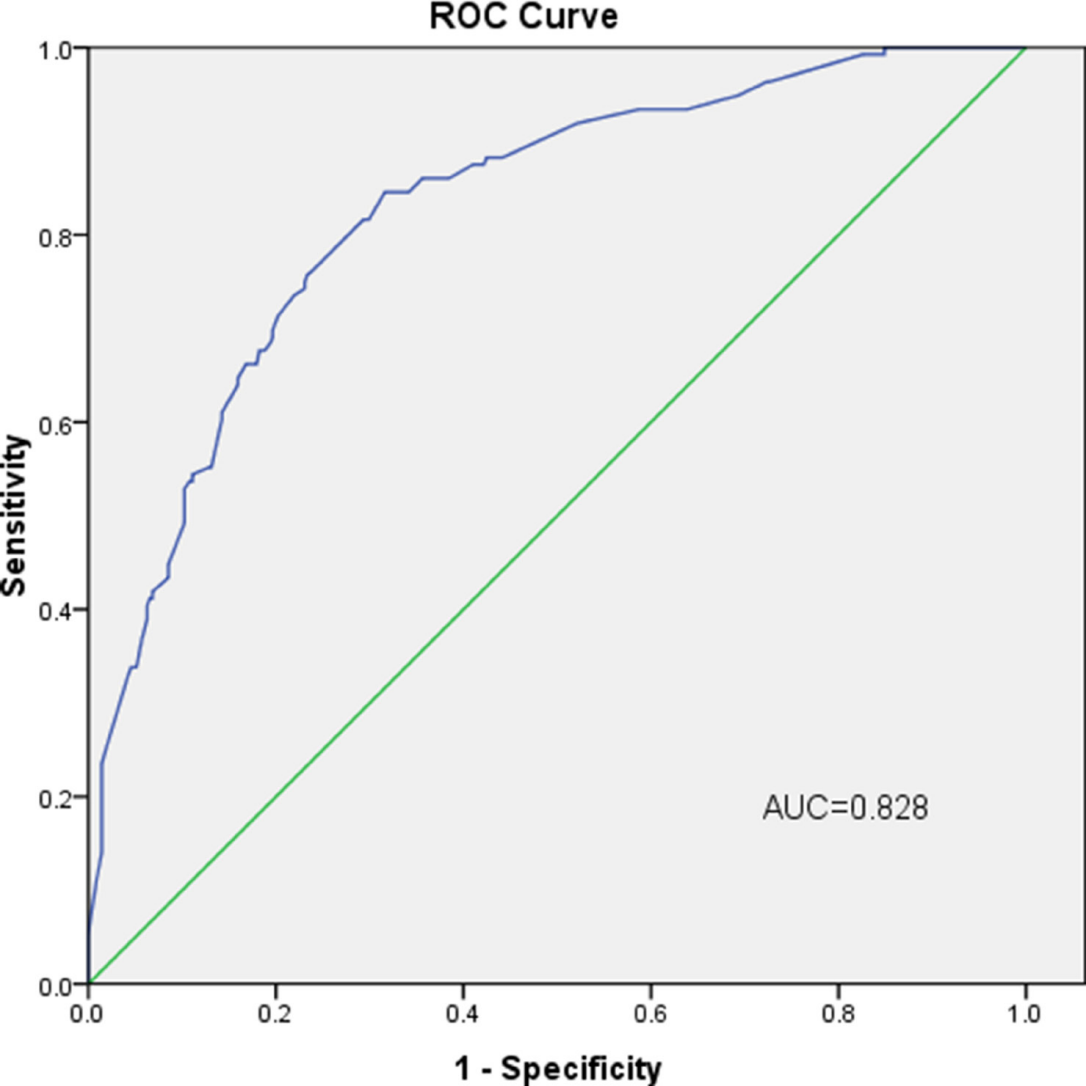
Internal validation using a ROC curve The AUC value is 0.828 (95% CI: 0.788–0.868).

**Figure 3:**
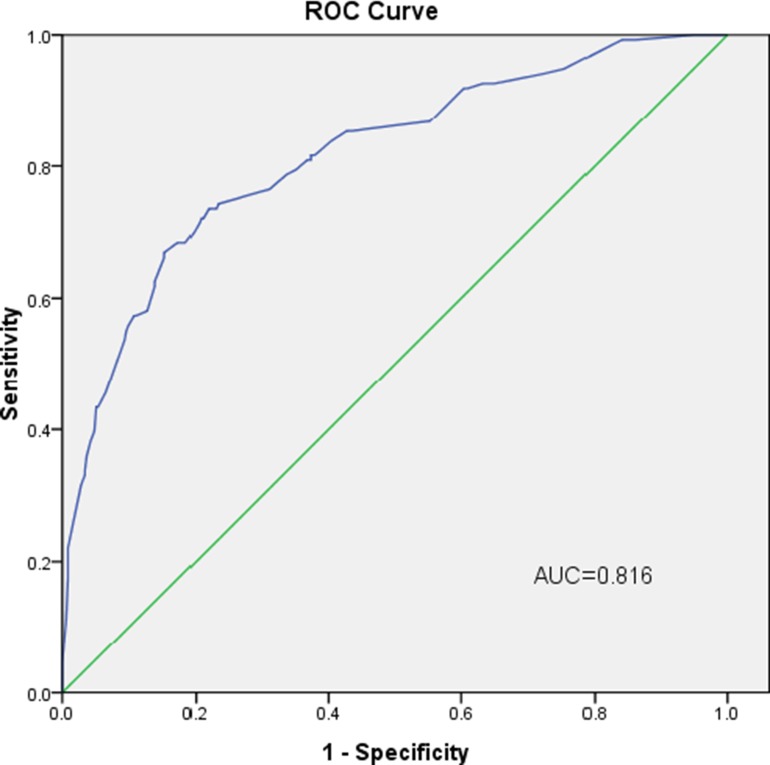
External validation using a ROC curve The AUC value is 0.816 (95% CI: 0.772–0.859).

**Figure 4:**
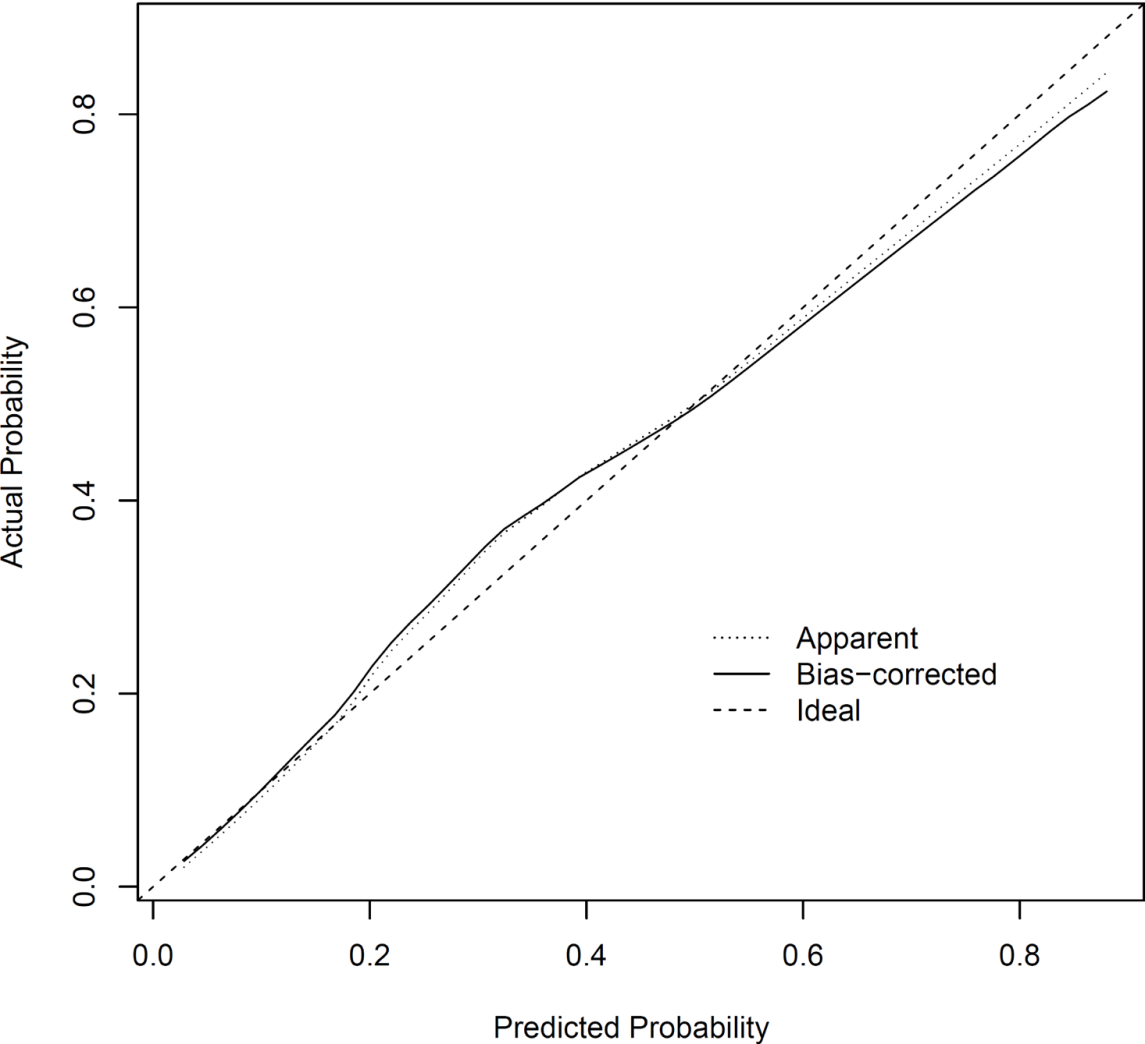
Calibration plot of the nomogram for the probability of L-2-ALNM

The internal receiver operating characteristic (ROC) curves in the modeling set (Figure 2) and external ROC in the validation set (Figure 3) were used to evaluate the nomogram. In the modeling and validation groups, the area under the curve (AUC) were 0.828 (95% confidence interval [CI]: 0.788–0.868) and 0.816 (95% CI: 0.772–0.859), respectively, which indicates a good predictive ability. Figure 4 showed that our bias-corrected curve was close to the ideal curve, which indicated that the predicted probability of L-2-ALNM was consistent with the actual L-2-ALNM risk. The *p* value in the Hosmer-Lemeshow test was 0.390, which confirmed a good fit for the model. The false-negative rate of the model was 7.41% at cutoff points of < 10% in the validation group (Table 4), and the predictive values between the modeling and validation groups were similar, which was conducive for further evaluation of the clinical usefulness of the model.

**Table 4:** Predictive values of the L-2-ALNM nomogram at different cutoff values in the validation group

Cut-off values(%)	No. of patients and percentage (%)	No. of falsenegative patients	False-negative rate (%)	Negative predictive value (%)
< 6	55 (11.29)	1	1.82	98.18
< 10	135 (27.72)	10	7.41	92.59
< 15	243 (49.90)	25	10.29	89.71

## DISCUSSION

Axillary lymph node status is a significant prognostic factor in patients with primary breast cancer and may plays a decisive role in deciding the therapeutic options [14–16]. With more in-depth research on breast cancer, some researchers believe that patients without axillary lymph node metastasis will not benefit from ALND [17]. According to a review by Hiken, surgical treatment of breast cancer patients should be divided into three groups [18]: (1) patients without clinically suspected axillary lymph node metastasis for whom axillary lymph node surgery can be completely avoided; (2) patients with limited positive nodes received only SLNB; and (3) patients with more positive axillary lymph nodes must perform ALND [18–19]. Although sentinel lymph node biopsy could be performed in patients with one or two metastatic lymph nodes, it could not be a reasonable treatment for patients with positive axillary lymph nodes confirmed preoperatively by clinical and imaging examinations. And the National Comprehensive Cancer Network (NCCN) guidelines showed that level 1/2 lymph node dissection were performed for the clinical stage I and stage II breast cancer patients with clinically positive nodes. So, our model could be more suitable for these patients with positive axillary lymph nodes confirmed preoperatively by clinical and imaging examinations. In addition, the treatment of omitting axillary lymph node dissection has not been practiced for the patients with one or two sentinel lymph nodes in China. Furthermore some studies showed that the incidence of postoperative complications such as upper arm lymphedema and upper limb numbness was remarkably lower in the patients receiving less lymph nodes dissection compared with patients who have more lymph nodes dissected. [8, 10] Therefore, level 1 ALND is a better option for patients with positive level 1 axillary lymph nodes but without L-2-ALNM. Therefore, how to use a noninvasive and reliable tool to accurately screen out these patients without L-2-ALNM become a crucial problem. In the era of individually and precisely tailored treatment, although accurate identification of patients without L-2-ALNM is still a challenging problem, predicting model of L-2-ALNM will become increasingly indispensable in formulating surgical regimens.

The model to predict L-2-ALNM in patients with L-1-ALNM has not previously been reported. In the present study, we developed a nomogram consisting of four independent factors relevant to L-2-ALNM in patients with L-1-ALNM. With the help of the model, an innovative approach of deciding to omit L-2-ALND offers several advantages compared with ALND. First, the elimination of unnecessary L-2-ALND can provide patients with better postoperative quality of life. Second, the model has the advantage of reducing the patient's medical expenses, such as reducing the cost of additional surgery, some diagnostic fees and lymph node pathology fees.

Predicting L-2-ALNM in level 1 node-positive breast cancer patients is critical to understand the prognosis of the patients, which directly determines the treatment of level 2 axillary lymph node. We retrospectively analyzed the clinical and pathological data of 974 patients with breast cancer, and established a predictive model to assess the risk of L-2-ALNM. With the help of the univariate and multivariate analyses, four independent predictors associated with L-2-ALNM were contained in the prediction model. They were tumor size, Ki-67 status, HER-2 status, histological grade, and number of PL-1-ALN. Many other studies have reported that tumor size and histological grade are risk factors for axillary lymph node metastasis [8, 20–22], consistent with the results we have studied. Ki-67 status as a predictor of axillary lymph node metastasis was reported by some researchers [23]. The AUC value of our modeling group was 0.828. We then applied an additional set of independent data to validate the model and gained an AUC value of 0.816, which indicated it was a model with excellent discrimination ability.

To the best of our knowledge, this is the first nomogram prediction model of level 2 axillary lymph node status in breast cancer patients with level 1 axillary lymph node metastasis reported in the English literature. In the light of the results, we believe that the proposed L-2-ALNM nomogram is both valid and useful in Chinese populations.

To further evaluate the clinical usefulness of the L-2-ALNM prediction model, we calculated the false-negative value of our model at probability cutoff values of < 6%, < 10%, and < 15% in the validation groups (Table 4). During the calculation, we observed that only the populations with a low risk of metastasis were exempted from undergoing L-2-ALND.

The study has reported that the overall false-negative rate of SLNB was 8.4% (0–29%) [24]. Therefore, we assume that most surgeons can accept a false-negative rate of 0–8%. In the validation group, when the cutoff values were < 6% and < 10%, the false-negative rates were 1.82% and 7.41%, respectively, which were lower than the false-negative rate of sentinel lymph node biopsy. The proportions of patients with L-2-ALNM rate of < 6% was 11.29% in the validation group. Meanwhile, the proportion of the patients with L-2-ALNM rate of < 10% was 27.72% in the validation group. This finding suggests that our prediction model could achieve the desired predictive effects. Hence, based on our prediction model, if the L-2-ALNM rates are < 6% or < 10%, these patients could be exempted from L-2-ALND based on the actual situation. This is particularly so for elderly patients with comorbidities, as ALND will not only increase the risk of surgery but also cause serious postoperative complications.

The methods of breast cancer treatment are continuously changing, and the management of axillary lymph nodes is also continuously evolving. As clinical research improves, the prediction of residual axillary lymph nodes will become increasingly important, as this will directly affect the development of treatment protocols for axillary lymph nodes. Our prediction model includes 4 common clinicopathological variables that can be obtained through routine examinations. Our predictive model had preferable predictive ability and clinical utility. As demonstrated in this study, the stability of our model allowed us to believe that the model was universally applicable to other Asian patient populations, although further validation in other populations is also desirable.

Apparently, the results of prediction models are more reliable than clinical speculation. Even though our prediction model shows better predictive power, but it still has limitations that need to be addressed. First, our model was developed based on a retrospective, single-center study and thus requires further validation in other research settings. In addition, only the patients with invasive ductal carcinoma were included in our study, which decreased the scope of application of the model.

In conclusion, we established an accurate, objective, and easy-to-use model to predict the probability of L-2-ALNM in the breast cancer patients with L-1-ALNM. In virtue of our nomogram, the surgeons could consider avoiding their L-2-ALND for low-risk patients with L-2-ALNM.

## MATERIALS AND METHODS

### Patients

Data were collected from 3444 breast cancer patients diagnosed with invasive ductal carcinoma who were treated between January 2010 and December 2014 at the Liaoning Cancer Hospital and Institute. Among them, a total of 34.2% (1179) patients had positive level 1 axillary lymph nodes. After excluding 205 patients with incomplete relevant information, a total of 974 patients were included in the study. Clinical data included in the analysis were the following: data on tumor size (categorized as T1 ≤ 2 cm, 2 cm < T2 ≤ 3 cm, 3 cm < T3 ≤ 5 cm, T4 > 5 cm), tumor location, immunohistochemistry (IHC) (estrogen receptor [ER], progesterone receptor [PR], human epidermal growth factor receptor 2 [Her-2], Ki-67), histological grade, age, menopausal status, and number of positive level 1 axillary lymph nodes (PL-1-ALN).

The inclusion criteria were as follows: 1) diagnosed with invasive ductal carcinoma, 2) operable primary breast cancer confirmed by core biopsy or open biopsy, 3) had positive level 1 axillary lymph nodes confirmed by pathology postoperatively, 4) underwent both radical excision of the primary tumor and ALND, 5) informed consent was obtained. Exclusion criteria were as follows: missing data, negative level 1 axillary lymph node confirmation by pathology, skin invasion, distant metastatic disease at diagnosis, not invasive ductal carcinoma, and receipt of neoadjuvant chemotherapy. The eligible patients were randomized 1:1 and divided into a modeling set (nomogram construction) and a validation set (nomogram validation). This study was reviewed and approved by the Ethics Committee of Liaoning Cancer Hospital and Institute.

### Treatment

Surgical treatment was performed on the basis of the Chinese breast cancer guidelines. Surgery included resection of the primary tumor and performance of level 1 and level 2 ALND. The histological status and quantity of level 1 and level 2 nodes were analyzed retrospectively.

### Data extraction

We utilized each of the following variables: tumor characteristics (histological grade; clinical tumor size; tumor location; and ER, PR, and HER-2 status; and Ki-67 status), age, menopausal status, and nodal status including level 1 and level 2 from surgical pathology reports after ALND.

### Pathologic evaluation

The Chinese breast cancer guidelines were used to evaluate surgical specimens. Tumors with ≥ 10% nuclear-stained cells were considered positive for ER and PR. Ki67 expression ≥ 20% was considered positive. HER-2 positivity was defined as a score of 3+ on IHC or amplification on FISH [13]. If a pathologist scored the IHC 2+, the HER-2 status was further investigated by FISH. In addition, the grade of breast cancer was determined by the Nottingham Histologic Score system.

### Statistical analysis

The chi-square test or Fisher's exact were utilized for the categorical data, and descriptive statistics were used for the within-group or between-group comparisons of independent samples. All statistical analyses were performed using SPSS 17.0 statistical software (SPSS Inc., Chicago, IL, USA) and R software (version 3.1.0). A two-tailed *P* value < 0.05 was considered statistically significant. All reported *P* values are two-sided.

Univariate analysis of the 10 factors described above was performed to decide which one was associated with L-2-ALNM. Variables that were statistically significant (*P* < 0.05) in the univariate logistic analysis in the modeling group were included in the multivariate logistic regression analysis, which was used to screen independent predictors for L-2-ALNM. Independent predictors (*P* < 0.05 in the multivariate logistic regression analysis) were used to construct a well-calibrated nomogram for predicting the probability of L-2-ALNM. An additional set of 487 Chinese patients in the validation group was used to validate the predictive model.

The performance of the model was quantified with respect to discrimination and calibration. First, discrimination is the predictor's ability to separate patients with different responses or events. The ROC curve was drawn, and the AUC was used to assess the discriminatory abilities of the model. Second, calibration is the agreement between the frequencies of observed outcomes and the probabilities predicted by the model. The calibration of the nomogram was performed internally by a calibration plot with bootstrap sampling (*n* = 1000). The nomogram was defined as well-calibrated if the calibration plot of the model fell along the 45-degree line. The Hosmer-Lemeshow test and visualize inspection of the plots were used to evaluate the calibration plot, and *P* > 0.05 indicated a good fit.

## Abbreviations

ALND: axillary lymph node dissection
L-2-ALNM: level 2 axillary lymph node metastasis
L-1-ALNM: level 1 axillary lymph node metastasis
PL-1-ALN: positive level 1 axillary lymph nodes
ROC: receiver operating characteristic
AUC: areas under the receiver operating characteristic curve
L-2-ALND: level 2 axillary lymph node dissection
ORs: odds ratios
CIs: confidence intervals
UOQ: upper outer quadrant
UIQ: upper inner quadrant
LOQ: lower outer quadrant
LIQ: lower inner quadrant
